# P071. Headache self-administered questionnaire in the waiting room, a useful and easy tool for headache centres

**DOI:** 10.1186/1129-2377-16-S1-A78

**Published:** 2015-09-28

**Authors:** Teresa Catarci

**Affiliations:** Azienda Sanitaria Locale ASL RMA, Rome, Italy

## Background

The diagnosis of headache relies mostly upon the patient's history, hence a detailed and time consuming medical history collection is required in the clinical setting. Second level headache centres of the Italian National Health Service often face the difficulty of a shorter availability of time. Therefore, we managed to elaborate a self-administered questionnaire to be filled in while the patients are in the waiting room, in order to gather the basic information prior to the visit.

## Methods

Most of the ICHD-II criteria for primary headache were transformed into questions (in Italian and English) formulated in such a way that they could be self-administered, easily understood, and quickly filled out. Patients who were consulting for headache for the first time were asked to fill in this 16 item self-administered questionnaire in an outpatient Neurology Clinic of the Italian National Health Service, located in the center of Rome (ASL RMA) between October 2014 and June 2015. We calculated adherence to the questionnaire and sorted out items that required to be further investigated during the visit.

## Results

One hundred and twenty patients were admitted to the outpatient clinic, 91 females and 29 males, mean age 45 years, range 18-80 years. Twenty-eight patients only, out of 120, did not fill in the questionnaire mostly because of language barriers. The item that needed further investigation during the visit was “describe what the headache is like” since most patients wrote uninformative descriptions like “bad” or “severe”. The item that went undescribed most times was “what makes your headache better” and “what makes your headache get worse”, while the best described item was “is there any symptom that comes together with the headache like tearing of your eyes, sickness or vomit, bright light and noise bother you?”

## Conclusions

This self-administered tool was well accepted by the patients. There are some important questions that still need to be further investigated during the visit, like the quality of pain and others that probably need the help of the headache specialist to be recalled. Nevertheless, we believe that administering a questionnaire in the waiting room is of help to the patient in order to better focus on informative items of the headache itself and to reduce possible visit-related anxiety. Last, but not least, it allows the headache specialist to gather headache history in a less time-consuming way.

Written informed consent to publication was obtained from the patient(s).

